# A new paradigm in management of frequent attenders to emergency departments with severe alcohol use disorder—A pilot study for assertive community treatment in Singapore

**DOI:** 10.3389/frhs.2022.1029455

**Published:** 2022-11-04

**Authors:** Charles Chia Meng Mak, Desmond Ren Hao Mao, Fahad Javaid Siddiqui, Alex Lim, Jayson Davamoni-Thomas, June Peiwen Tang, Rozinah Bachik, Charis Wei Ling Ng, Gomathinayagam Kandasami, Cheng Lee

**Affiliations:** ^1^National Addictions Management Service, Institute of Mental Health, Singapore, Singapore; ^2^Acute & Emergency Care, Khoo Teck Puat Hospital, Singapore, Singapore; ^3^Prehospital and Emergency Care Research Center (PERC), Health Services and Systems Research, Duke-NUS Medical School, Singapore, Singapore; ^4^Population Health & Community Transformation, Khoo Teck Puat Hospital, Singapore, Singapore; ^5^Clinical and Forensic Psychology Service, Ministry of Social and Family Development, Singapore, Singapore; ^6^Education Office, Institute of Mental Health, Singapore, Singapore

**Keywords:** assertive community treatment, alcohol-related frequent attenders, frequent attenders, alcohol, emergency departments

## Abstract

**Introduction:**

A majority of frequent users of emergency medical services in Singapore present with alcohol-related problems. These patients are known to engage poorly with traditional addiction services and frequently attend Emergency Departments (EDs) instead, resulting in high healthcare burden. Assertive Community Treatment (ACT) is an alternative intervention to traditional addiction management. ACT involves community visits with focus on holistic care and harm-reduction.

**Materials and methods:**

We conducted a prospective before-and-after cohort study at the major tertiary center for addiction disorders in Singapore. The main objective was to evaluate effectiveness of ACT in reducing alcohol-related attendances at EDs nationwide. Socio-demographics, alcohol-related ED attendances, and the Christo Inventory for Substance-misuse Services (CISS) scores were collected for the patients recruited from April 2018 to March 2019. Descriptive analyses and the Wilcoxon Signed-Rank Test were performed.

**Results:**

All 14 patients were male with a mean age of 55 years. There was a significant 45.3% reduction in average alcohol-related ED attendances from 6.8 (range 3–22, median 5.5) in the pre-intervention 6-month period, to 3.7 (range 0–28, median 1.5) in the post-intervention 6-month period (*Z* = −2.244, *p* = 0.025). CISS scores showed significant improvement from a pre-intervention median of 13.5 (range 9–16) to a post-intervention median of 6.5 (range 1–10, *p* = 0.001), corresponding to reduction in alcohol-related problem severity.

**Conclusion:**

This pilot study suggests that ACT can be effective in reducing alcohol-related ED attendances and alcohol-related problem severity in patients with AUD who frequently attend ED. A multicenter, prospective study using ACT for such patients across four hospitals in Singapore is currently underway.

## Introduction

The majority of patients with Alcohol Use Disorder (AUD) do not receive appropriate addiction treatment (1). Those who do present for addiction treatment are known to drop-out prematurely, with drop-out rates of up to 75.0% by their fourth session (2). Local data from the National Addictions Management Service (NAMS), Singapore, is in keeping with this trend, with up to 75.0% of patients with AUD dropping out of counseling by their third session.

In lieu of attending addiction treatment, a proportion of patients with AUD will present at high frequency to Emergency Departments (ED) for alcohol-related problems. In Singapore, 51.3% of patients aged < 65 years, who were frequent users of emergency medical services, presented with alcohol-related problems (3). Data on ED attendances between the period of 2007–2016 shows a steady increase in alcohol-related ED utilization, with 4,433 alcohol-related visits in 2016 compared to just 2,236 in 2007. The total number of alcohol-related attendances also rose at a higher rate in comparison to non-alcohol attendances by 36.8% (4).

Patients with AUD who frequently present to ED are known to suffer from poor physical and mental health, and high levels of unmet social needs. These features perpetuate alcoholism and poor engagement with addiction treatment services (5). Frequent attendances at ED translate to financial burden upon healthcare and public expenditure, as well as intangible costs such as increased ED wait-times. In the United Kingdom, around £3.5 billion (SGD6.1 billion) was spent per year on healthcare costs due to alcohol misuse (6). In the United States, alcohol misuse leads to wastage of an estimate of US$184.6 billion (SGD257.4 billion) on healthcare, business, social, and criminal justice costs (7).

For patients with AUD who frequently attend ED, the conventional expectation for attendance at addiction treatment clinics and an abstinence outcome may be difficult to achieve. An alternative proposal is for treatment to be conducted assertively in community settings, with a focus on holistic care along a harm reduction approach to reduce alcohol-related morbidity (8).

Assertive Community Treatment (ACT) is an established model of care developed during the 1970s for patients with severe mental illness such as schizophrenia, with a tendency for frequent hospital re-admissions. Patients were seen in the community and provided intensive case management and follow-up (9, 10). Subsequent dramatic reductions in hospital re-admission rates and savings in public healthcare costs were noted (11).

The ACT model has been applied in the management of patients with AUD who frequently attend ED in the United Kingdom. In the ACTAD trial, Drummond et al. demonstrated improvements in patient engagement with addictions treatment, reduced alcohol use and unplanned healthcare costs (12). An ACT service in Salford, United Kingdom, led to a 66.0% reduction in unplanned hospital admissions and 60.0% reduction in emergency attendances 3 months after intervention (13). This reduction translated into healthcare savings of £606,675 (SGD1.1 million) in its second year of service (14).

**Figure 1:**
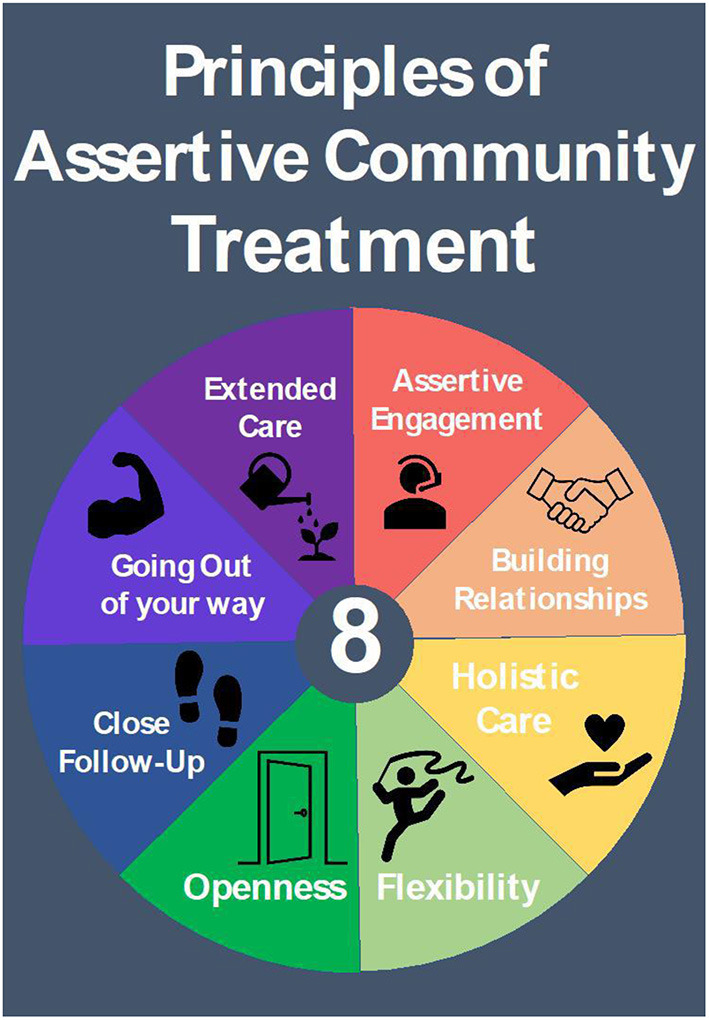
Principles of assertive community treatment.

In April 2018, the National Addictions Management Service (NAMS) conducted a pilot, real-world, implementation study on ACT intervention for patients with AUD who frequently attend ED in Singapore. This involved a departure from the model of care provided at the NAMS clinics. In the NAMS model of care, patients are expected to regularly attend clinic sessions for medication and counseling to work toward abstinence. In contrast, ACT intervention took place in the community and worked toward reducing alcohol usage and improving health and psychosocial functioning. The ACT intervention was based upon the model outlined in the ACTAD trial (12). The guiding principles of ACT are in Figure 1. Details of ACT intervention can be seen in Annex 1.

To the best of the authors' knowledge, this is the first service of its kind to be implemented in Asia.

## Materials and methods

### Study setting

The study was conducted in Singapore, a Southeast Asian Island city-state nation of 5.7 million people (Singapore Department of Statistics 2021). NAMS of the Institute of Mental Health is the only major tertiary treatment facility for addiction disorders in Singapore. From 2018 to 2020, NAMS received 23,715 patient referrals, with one third of this number being for alcohol misuse. Treatment at NAMS is clinic-based and provided primarily by a team of doctors and counselors (15). All public hospitals in Singapore routinely refer patients with AUD to NAMS for further management.

### Study design

We conducted a single-site, prospective, pragmatic, real-world, implementation, before-and-after cohort study on the impact of the ACT intervention on patients with AUD who frequently attend ED patients. The main objective was to demonstrate that ACT intervention was effective in reducing alcohol-related attendances at EDs nationwide.

### Patient selection

Inclusion criteria were as follows:

- Primary problem of AUD
- Unwillingness to receive treatment at a NAMS clinic
- Six or more alcohol-related attendances to any Emergency Department (ED) across Singapore in the preceding 12 months.

Patients were excluded if they were diagnosed with Antisocial Personality Disorder or Dementia, or misused illicit substances within the preceding 12-month period.

Alcohol-related ED attendances were defined as being unplanned and for acute sequelae of alcohol use. Examples of alcohol-related ED attendances included visits due to acute intoxication, falls from intoxication or acute alcohol-related medical conditions such as alcoholic gastritis or hepatitis. Chronic conditions such as symptomatic ascites from liver cirrhosis and unrelated conditions requiring ED visits were excluded. This classification was conducted by a single psychiatrist.

### Patient recruitment

Patients were recruited between April 2018 and March 2019. Doctors and counselors at NAMS clinics identified AUD patients with frequent ED attendances, and referred them to the ACT pilot service to determine suitability for the study.

### Intervention

Patients underwent ACT intervention. Case workers assertively engaged patients with AUD who frequently attend ED, in accordance to ACT principles (Figure 1). Patients were seen in community settings (at home, or at communal areas typical of Singapore, such as hawker centers and void decks) over a period of 6 months. Each session lasted ~1 h and occurred at the following frequency; weekly for months one and two, every other week for months three and four, and once-monthly for months five and six. Due to the assertive nature of engagement, patient visit defaults were uncommon, but would be rescheduled if it occurred. During the course of the 6 months of follow-up, patients were progressively linked up with community resources such as Social Service Agencies (SSAs), addiction drop-in centers and support groups, for the purpose of step-down care at the cessation of ACT.

A framework for intervention was utilized to ensure treatment fidelity (Table 1). Upon each patient interaction, case workers explored for problems across four life domain categories; Medical, Psychological, Social, and Alcohol. The Christo Inventory for Substance-misuse Services (CISS) was obtained by case workers at monthly intervals to examine the impact of alcohol on patient's health, psychosocial and occupational functioning (16). Problems across the four life domain categories and CISS scores were discussed with a psychiatrist during the weekly multi-disciplinary team meetings.

**Table 1:** Framework for assertive community treatment.

i. Exploring for problems across four life domains (medical, psychological, social, alcohol). ii. Monthly CISS^*^ scores. iii. Weekly multi-disciplinary team meetings involving a psychiatrist. iv. Community visit frequency. a. Months 1 and 2—weekly. b. Months 2 and 3—every other week. c. Months 4 and 6—monthly.

* CISS, Christo Inventory of Substance-misuse Services.

This ACT service did not provide for 24-h availability, but case workers were contactable during working hours for any queries in relation to health and psychosocial needs. A fee waiver for the service was applied as it was surmised that such patients would have difficulty in affording treatment fees.

### Data collection and outcome measures

Socio-demographic information (age, gender, ethnicity, marital status, living arrangements, and employment status) and the primary outcome measure of the number of alcohol-related ED attendances over a 6-month period before and after commencement of ACT were extracted from the patient's medical records by a single trained medical abstractor.

The secondary outcome measure was in relation to the impact of ACT on the patient's health, psychological and occupational functioning. This outcome was measured using the Christo Inventory of Substance misuse Services (CISS), with a total score obtained at monthly intervals for 6 months. CISS is a validated evaluation tool completed by drug/alcohol service workers from direct patient interviews, whereby scores are obtained by exploring for problems over the past month in the following 10 domains; social functioning, general health, sexual/injecting risk behavior, psychological functioning, occupation, criminal involvement, drug/alcohol use, ongoing support, compliance, and working relationships. Each domain is scored on a three-point scale of problem severity–0 for none, 1 for moderate, and 2 for severe. A total score from 0 to 20 is calculated, with a higher total score being indicative of poorer outcomes. For outpatient alcohol service groups, a CISS total score of 0–4 indicates low problem severity, while 5–11 indicates average problem severity, and 12–20 indicates high problem severity (16).

Descriptive analyses of the socio-demographics were performed. The Wilcoxon Signed-Rank Test was used to compare the changes in alcohol-related ED visits and CISS scores. The data was analyzed using IBM SPSS software version 27.

Ethical approval was obtained from the Domain Specific Review Board of the National Healthcare Group, Singapore.

## Results

**Figure 2:**
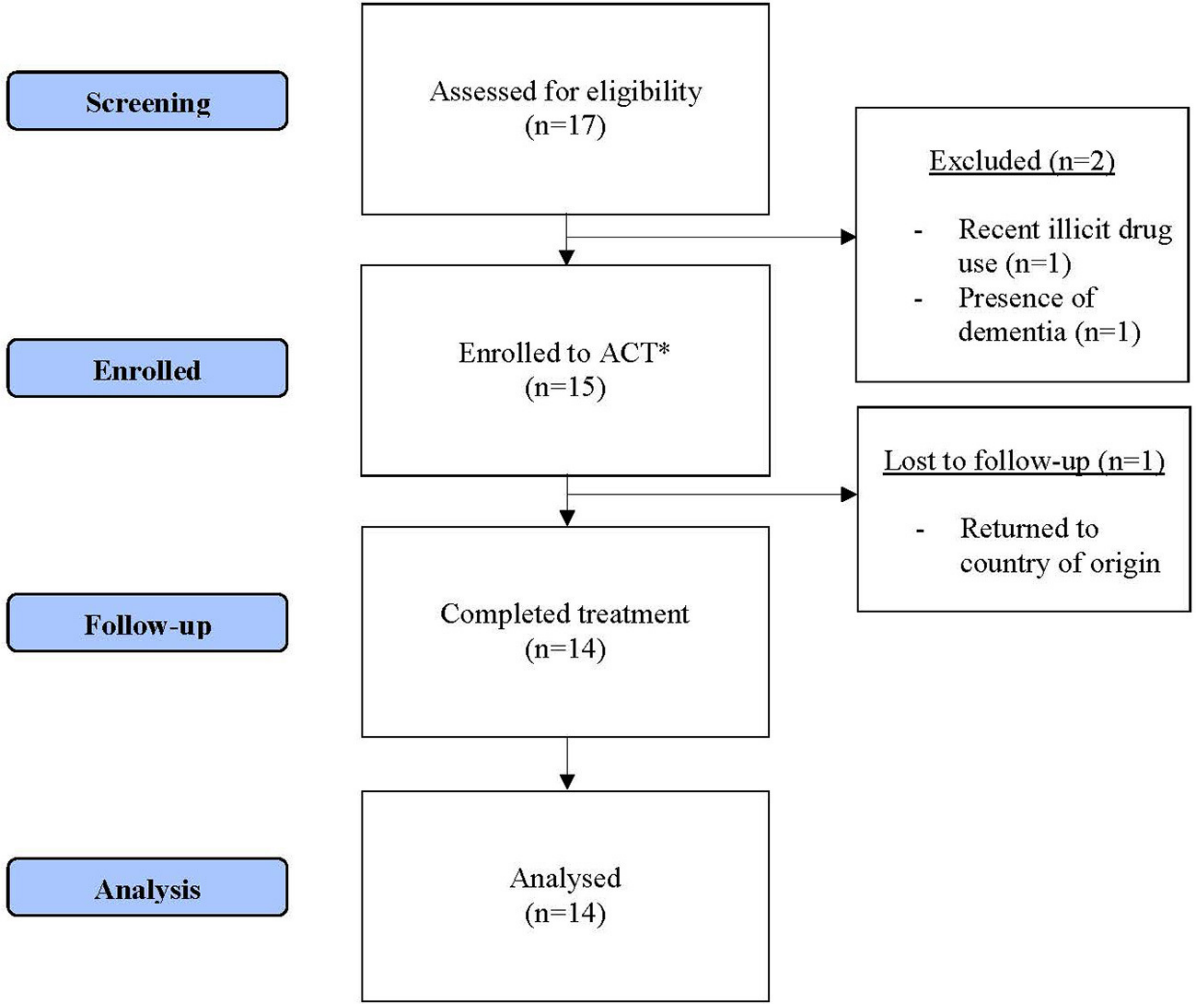
Recruitment flow. *ACT, assertive community treatment.

A total of 17 patients were referred for this study (Figure 2). Two patients were excluded as they did not meet inclusion/exclusion criteria. The remaining 15 patients were provided ACT. One patient was lost to follow-up as he returned to his country of origin. Missing data was minimal. Individual domains of a single patient's baseline CISS score were missing. We used information from the second visit's CISS score to impute into the baseline domain scores.

The socio-demographics of the 14 patients who were provided and completed ACT are presented in Table 2. All patients were male, with a mean age of 55.3 years old. The majority of the sample were of Chinese or Indian ethnicity, unemployed, and either living alone or destitute.

**Table 2:** Socio-demographics of assertive community treatment patients (*n* = 14).

		**Range**
**Age (years)**		
Mean (SD)	55.3 (9.3)	37–76
Median (IQR)	54 (50–59)	
	**No**.	**%**
**Gender**		
Male	14	100.0
**Ethnicity**		
Chinese	6	42.9
Malay	2	14.3
Indian	5	35.7
Others (Sikh)	1	7.1
**Marital status**		
Single	3	21.4
Married	4	28.6
Divorced/separated	6	42.9
Widowed	1	7.1
**Living arrangements**		
Alone	5	35.7
Family (spouse/children/parents/siblings)	5	35.7
Destitute/homeless	4	28.6
**Employment status**		
Employed	2	14.3
Unemployed	12	85.7

SD, Standard deviation.

IQR, Interquartile range.

There was a reduction in alcohol-related ED attendances by 45.3% from 95 visits pre-ACT to 52 visits post-ACT. An average number of 6.8 alcohol-related ED attendances per patient (range 3–22, median 5.5) in the pre-intervention 6-month period, dropped to 3.7 (range 0–28, median 1.5) in the post-intervention 6-month period (*Z* = −2.244, *p* = 0.025). More details may be found in Annex 2.

Three patients did not have a decrease in alcohol-related ED attendances, of which two had comparatively minor increase in usage of ED and one with no change compared to the pre-intervention period. All three patients were of Chinese ethnicity and were unemployed.

CISS scores showed significant improvement from a pre-intervention median of 13.5 (range 9–16) to a post-intervention median of 6.5 (range 1–10, *p* = 0.001). This corresponded to a reduction in the level of problem severity, from a high level at pre-intervention, to an average level at post-intervention. All patients showed improvement in their CISS scores. Twelve patients showed improvement in their problem severity after 6 months—three patients from high to low severity, eight patients from high to average severity, and one patient from average to low severity. Two patients remained at average problem severity. Besides the criminal involvement domain, the patient cohort showed improvements in almost all CISS domains at the end of the 6-month intervention (Table 3).

**Table 3:** Christo inventory for substance-misuse services individual domains and emergency department total visits (*n* = 14).

**Domains**	**Improvement (No. of patients)**	**Worsening (No. of patients)**	**Nil change (No. of patients)**	*p*-value^*^
Social functioning	7	1	6	0.021
General health	10	1	3	0.008
Psychological	7	0	7	0.008
Occupation	6	0	8	0.024
Criminal involvement	3	1	10	0.317
Alcohol use	5	0	9	0.038
Ongoing support	10	1	3	0.005
Compliance	11	0	3	0.002
Working relationship	9	0	5	0.004
Overall CISS score	14	0	0	0.001
Emergency department visits	11	1	2	0.025

* Wilcoxon Signed-Rank Test.

Pearson's correlation between change in CISS score and change in ED attendance was 0.456 (*p* = 0.101).

## Discussion

Our pilot study showed that in this patient population, ACT was associated with significant reduction in alcohol-related ED utilization and improvement in CISS total scores.

The primary outcome was achieved, with an overall 45.3% reduction in the average number of alcohol-related ED attendances per patient, at cessation of ACT. The majority of the group responded positively to ACT, with 11 patients experiencing a drop in alcohol-related ED attendances. Of these, most were of dramatic reductions and four patients had complete cessation in alcohol-related ED usage.

Post-intervention, the median total CISS score was significantly reduced, with categorical reduction from an initial high problem severity to average problem severity. This indicates improvement in overall health, psychosocial and occupational functioning in the group.

A recent systematic review (17) on various interventions targeting ED frequent attenders showed that the magnitude of subsequent decrease in ED usage ranged from 13.2 to 43.0%. In contrast, the single UK-based ACT study utilizing the same primary outcome showed a more impressive reduction in ED usage of 59.4% (13). The magnitude of reduction in ED attendances achieved in our study is consistent with research on ACT in the UK, and this finding deserves to be replicated in a larger scale.

Our study's patient population appears to be characterized by severe alcoholism and socio-economic difficulties. The majority of the patient group was unemployed and lacked ongoing support, with high rates of living alone, divorce and/or separation being observed. The presence of problems across health and psychosocial domains was noted, with the median CISS score corresponding to high problem severity at the start of treatment.

28.6% of the patient group were rough sleeping and were classified as destitutes. A 2019 study found that around 1,000 individuals rough sleep in Singapore (18). Our patient group had a disproportionately high number of destitutes, suggesting that there was an association between alcohol usage, ED visits and rough sleeping. In keeping with Maslow's hierarchy of needs, rough sleeping and other severe social stressors explains the difficulty for this patient population in adhering to traditional clinic-based addiction treatment. In contrast, presenting at EDs allow for immediate needs to be met, such as shelter and support. This lends further credence to ACT as a more appropriate service for such patients, being conducted assertively in the community with a focus on flexible and holistic patient-led care.

It is encouraging to note that patients improved in almost all CISS domains. Patients with AUD who frequently attend ED are known to suffer from severe and intractable alcoholism, and for whom recovery can be a lengthy journey. Patient engagement with treatment services and step-down to community resources is key to facilitating recovery over the long-term. Improvement in patient engagement may help to explain the effect of ACT on reducing ED attendances, especially in light of demographic findings suggesting isolation and low levels of support in this group.

The relationship between change in CISS scores and change in ED attendance has not been explored in literature. Intuitively, we believe that patients with improvement in problem severity should have fewer alcohol-related ED visits. Our study showed that change in CISS scores had moderate positive correlation with change in ED attendance. While this finding did not reach significance (*p* = 0.101), this could due to a type II error and needs to be explored in larger studies.

Reduction in alcohol-related ED attendances was not achieved for three patients in the pilot group. However, all three patients had improvement in total CISS scores at the end of ACT intervention, with two having their initial high problem severity reduced to an average problem severity. This suggests that ACT yields benefits even for patients who do not respond with reduction in alcohol-related ED usage. A larger sample is required to observe if this is a consistent trend. It should be noted that while ED visits by such patients are less likely to result in hospitalization (3), they may suffer from chronic complications related to alcohol requiring urgent usage of ED.

### Areas for future research

As a result of findings from this novel pilot study, a larger prospective interventional study using ACT to treat patients with AUD who frequently attend ED is currently underway. This larger study involves four hospital study sites across the nation and has the potential to treat all such patients in Singapore. Active data collection on costs incurred by public services is embedded into its design, and the effect of loneliness and its relation to ED attendances will be explored through loneliness measurement scales.

Calculation of overall cost savings for the healthcare system is a challenging task. The cost of an ED attendance at a public hospital in Singapore is rounded up to a standard fee following a heavy subsidy, but it is likely that patients with AUD who frequently attend ED incur higher true costs. It can be postulated that costly procedures will often be required, such as in radiological imaging following alcohol-related falls and injuries. Involvement of ambulance and police force utilization in conveying them to ED sites is not unusual. As such patients face socio-economic problems, it is unlikely that the already subsidized fees are ever recuperated in the vast majority of instances. Therefore, ACT has the potential for overall cost savings for the Singapore healthcare system. The same multicenter study with active data collection on cost utilization will help to answer this question.

Studies on ACT for mental disorders have suggested that reduction in readmission rates are maintained after cessation of the ACT intervention (19). It would interesting to note for similar, sustained reduction in ED attendances following the 6-month ACT intervention.

### Strength and limitations

Being the first of its kind in Asia, the main strength of this study is its novelty. ACT intervention involved a departure from the clinic-sited and counseling-based care typical for addiction disorder management in the region. It was conducted by the main addiction disorder management service in Singapore, which is not limited to geographic service boundaries.

Our study is subject to the usual limitations of before-and-after cohort studies. One patient was lost to follow-up, but this accounted to < 20% of the group. A large population group is favorable for a prospective cohort study, but only a small number was recruited as this was a pilot intervention. The use of counselors for ACT from the only major tertiary referral center for AUD limit generalizability to other settings.

## Conclusion

In this prospective, real-world, implementation, before-and-after pilot study on patients with AUD who frequently attend ED, ACT was shown to be associated with a decrease in alcohol-related ED usage and an improvement in functioning in health, psychosocial and occupational domains. An ambitious, multicenter study involving ACT intervention is underway to treat this nationwide patient population.

## Data availability statement

The raw data supporting the conclusions of this article will be made available by the authors, without undue reservation.

## Ethics statement

The studies involving human participants were reviewed and approved by Domain Specific Review Board of the National Healthcare Group, Singapore. Written informed consent for participation was not required for this study in accordance with the national legislation and the institutional requirements.

## Author contributions

CM, RB, JD-T, AL, JT, GK, and CL: conception and design. CM and CN: data curation. DM, FS, CN, and CM: analysis and interpretation of data. CM, DM, AL, JT, and RB: writing (original draft preparation). All authors: writing (reviewing and editing). All authors have read and approved the final article.

## Funding

DM was supported by the Alexandra Health Fund Ltd through the Research Investigator-Scientist Enabler (RISE) Grant.

## Conflict of interest

The authors declare that the research was conducted in the absence of any commercial or financial relationships that could be construed as a potential conflict of interest.

## Publisher's note

All claims expressed in this article are solely those of the authors and do not necessarily represent those of their affiliated organizations, or those of the publisher, the editors and the reviewers. Any product that may be evaluated in this article, or claim that may be made by its manufacturer, is not guaranteed or endorsed by the publisher.

## Annex

**Annex 1:** Details of assertive community treatment intervention.

1. **Assertive engagement**—assertively seeking contact with patients in home and community settings.
2. **Building relationships**—building rapport, maintaining contact, working with and supporting family members.
3. **Holistic care**—focusing on health, psychosocial and occupational needs.
4. **Flexibility**—working flexibly according to patients' goals.
5. **Openness**—being open with care plans and intended outcomes.
6. **Close follow-up**—maintaining a low staff-to-patient ratio to allow for effective case management and short intervals between reviews.
7. **“Going out of your way”**—stepping outside of professional roles and going the extra mile for patients.
8. **Extended care**—intensive case management and establishing links with community services and resources, to facilitate long term support.

**Annex 2:** Individual patient outcomes.

	**Total CISS*score/Severity category *pre-intervention***	**Total CISS score/Severity category *post-intervention***	**Alcohol-related ED∧attendances -*6 months pre-intervention***	**Alcohol-related ED attendances -*6 months post-intervention***	**Change in alcohol-related ED attendances (No. /proportion)**
Patient 1	14/High	1/Low	5	1	Reduced by 4/80.0%
Patient 2	15/High	2/Low	9	1	Reduced by 8/88.9%
Patient 3	11/Average	9/Average	3	1	Reduced by 2/66.7%
Patient 4	14/High	4/Low	8	2	Reduced by 6/75.0%
Patient 5	16/High	9/Average	6	0	Reduced by 6/100.0%
Patient 6	13/High	8/Average	6	8	Increase by 2/33.3%
Patient 7	9/Average	8/Average	3	3	Nil change = 0/0.0%
Patient 8	12/High	7/Average	10	0	Reduced by 10/100.0%
Patient 9	12/High	7/Average	22	28	Increased by 6/27.3%
Patient 10	15/High	10/Average	5	4	Reduced by 1/20.0%
Patient 11	14/High	10/Average	3	2	Reduced by 1/33.3%
Patient 12	12/High	6/Average	8	0	Reduced by 8/100.0%
Patient 13	14/High	9/Average	3	2	Reduced by 1/33.3%
Patient 14	9/Average	1/Low	4	0	Reduced by 4/100.0%
	**Median** **13.5/High**	**Median** **7.5/Average**	**Total** **95**	**Total** **52**	**Reduced by 43/45.3%**

*CISS, Christo Inventory for Substance-misuse Services. ∧ED, Emergency Department.
